# Oxyphosphate Fillings Not Injurious

**Published:** 1900-04

**Authors:** G. S. Junkerman

**Affiliations:** Cincinnati.


ARTICLE VIII.

### OXYPHOSPHATE FILLINGS NOT INJURIOUS.

PROF. G. S. JUNKERMAN, CINCINNATI.

That pulps die under oxyphosphate cannot be denied. That some have died under oxyphosphate that would have survived some other kind of filling material is also not debatable; but I do not believe that in a perfectly typical case of a thin perfect stratum of dentine being covered with a perfectly mixed cement, death of the pulp will occur.

There are many circumstances that alter the effect of cement used in deep seated caries. Many times from imperfect diagnosis, due either to the carelessness of the operator, or often to our inability to determine whether there is or is not an actual exposure of the pulp, the results will prove satisfactory. If an imperceptible exposure exists, oxyphosphate will accomplish destruction of the pulp as readily as any other material placed upon it. Thus I do not believe that any pulp that is actually exposed can be preserved alive for any length of time under any kind of filling material.

I do not believe, however, that oxyphosphate should receive the censure that such results receive, where deep-seated caries has advanced to an actual exposure of the pulp, whether that exposure be perceptible or not. Many practitioners jump at conclusions exactly in cases of such a nature. If a perfect stratum of dentine exists between the pulp and all probability of actual exposure is removed, and if the cement is properly mixed, I do not believe that such a pulp will be injured. Take three typical cases in which a perfect stratum of dentine exists. Introduce in the first case the cement properly mixed, free from all superfluous acid. In the second case varnish with shellac the intervening stratum, as a precautionary measure, and introduce perfectly mixed cement. In the third case introduce cement that is not properly mixed, upon either a varnished or unvarnished surface, there being a surplus of acid present; the first two cases will be successful, the last will prove fatal as to the vitality of the pulp.

It is a surprising fact how few practitioners mix cement properly. Many pulps are destroyed by the use of this filling material when its use should have preserved them. I could give a whole hour's discourse on the subject of mixing cement. All cements have their peculiarities, and before any one of them is used, a perfect comprehension should be had of all its peculiarities. I have no doubt but that we would receive less criticism in the use of cement in the deep-seated cases of caries, if every operator had full knowledge of how to use cement and how to mix it. If we cannot use cement in deep-seated caries, then its chief function is gone as a non-irritant, non-conducting filling material. We must relegate it then strictly to the use of setting crowns and bridge work. But even in cases of this kind, its usefulness would be limited, because we would not feel justified in using it in many cases where crowns and bridges are set upon vital teeth. Cement does not fulfill all requirements, but we must believe that the percentage of complaints could be much reduced if it were properly used.

The question is asked, how do cement fillings destroy pulps? I would answer this question by stating that, in my judgment, pulps are destroyed by the acid from the cement acting as an irritant to the pulp. We have no doubt established the fact that fluids permeate the dental tubuli, and that when there is a change in the chemical reaction there also occurs an osmosis outward or inward. The natural condition of the pulp is to be of an alkaline reaction. When you place fluid on the other side of the dental tubuli you produce an inward osmosis, and I feel thoroughly convinced that should the conditions of the pulp chamber be examined in a case where there has been a surplus of acid placed over the pulp, we would find phosphoric acid within the pulp chamber. The death of the pulp is accomplished no doubt from the effects of the phosphoric acid as an irritant, and the endeavors and struggles of the pulp to throw off this irritant, as would occur in any other part of the human body where an irritant were present.—*Items of Interest.*

